# Schistosomiasis Consortium for Operational Research and Evaluation: Mission Accomplished

**DOI:** 10.4269/ajtmh.19-0838

**Published:** 2020-05-12

**Authors:** N. Robert Bergquist

**Affiliations:** Swiss Tropical and Public Health Institute (Swiss TPH), Basel, Switzerland

## Abstract

The Schistosomiasis Consortium for Operational Research and Evaluation (SCORE), a program focusing on schistosomiasis control in sub-Saharan Africa between 2008 and 2019, investigated ways to improve coverage and efficacy of ongoing chemotherapy programs and concluded that because of continued transmission, mass distribution of praziquantel cannot eliminate the disease without complementary control activities. Schistosomiasis Consortium for Operational Research and Evaluation’s activities comprised large-scale, multicountry field studies comparing various mass drug administration strategies and some specific research avenues, such as assessment of high-sensitivity diagnostics, identification of hotspots, quantification of the role of the snail host, predictive modeling, and changes in schistosome population genetics under drug pressure. The discoveries made and the insights gained regarding cost-effective strategies for delivering preventive chemotherapy should assist policy makers to develop guidelines for the control and ultimate elimination of schistosomiasis.

## INTRODUCTION

The findings of the Schistosomiasis Consortium for Operational Research and Evaluation (SCORE), presented in this 15-article supplement in the *American Journal of Tropical Medicine and Hygiene*, provide tangible support that the long struggle against schistosomiasis will end successfully and well before the 200-year anniversary of Theodor Bilharz’s 1851 discovery of the adult worm in Egypt.^1,2^ More than 60 years were to pass before freshwater snails were shown to be part of the parasite’s life cycle^3^ and then another 50 years until the synthesis of the first efficacious drugs.^4,5^ In Japan, the process took as long, but developed in the opposite direction, with the disease described first (in the late 1840s), and the parasite not found until almost 70 years later.^6,7^ Altogether, including Sambon’s discovery of *Schistosoma mansoni* in 1907,^8^ more than 50 years passed between Bilharz’s first publication and the full historical account, replete with the three main schistosome species.^9^ The time was then ripe for efforts to control the disease, the early years of which have been brilliantly chronicled by Jordan^10^ and Fenwick.^11^

The twists and turns of schistosomiasis control efforts in various parts of the world came together in the late 1970s thanks to the advent of the drug praziquantel (PZQ)^12^ and its validation in the field.^13^ However, what made chemotherapy useful in practice was its dramatic drop in cost,^11^ and the welcome PZQ donation through the WHO by the German pharmaceutical firm Merck KGaA (Germany), enabling mass drug administration (MDA). In 1984, WHO recommended PZQ for schistosomiasis morbidity control^14^ and eventually developed a “roadmap”^15^ setting targets for elimination of the world’s most neglected tropical diseases (NTDs), including schistosomiasis, an initiative endorsed in 2012 under the “London Declaration on NTD,” a partnership comprising endemic countries, major donor organizations, and large pharmaceutical companies.^16^

## THROUGH THE LOOKING GLASS

Historically, four countries stand out with respect to long-term schistosomiasis control: Japan, which eliminated the disease as early as 1977, so far the only country to actually achieve this^17^; Egypt, where attempts began as early as 1922^18,19^; China, where a national control program has been active without interruption since 1958 under the slogan “political leadership, government support, and mass involvement”^20,21^; and Brazil, launching “SUperintendência de CAMpanhas de Saúde Pública” to control several NTDs in 1975, a program still operating through various governmental institutions.^22^ The early years of PZQ use has been chronicled in an account^23^ commissioned by WHO and supported by the Edna McConnell Clark Foundation (EMCF).

The first major project attempting large-scale PZQ distribution in endemic areas was the German Technical Cooperation^24^ working in selected African countries between 1982 and 1992. The resulting decline in schistosomiasis prevalence^25^ was heralded as a major advance, although this approach was later questioned, as prevalence returned to previous levels when not followed up.^26^ In Egypt, the Schistosomiasis Research Project (SRP), active between 1988 and 1998 with support from the United States Agency for International Development and the Ministry of Health,^27^ represented a mix of broad research support and practical control activities. Schistosomiasis Research Project left behind an effective strategy that eventually introduced the use of PZQ without prior diagnosis in endemic areas.^11^ In China, the World Bank Loan Project (WBLP) worked with the national control program in a way similar to SRP in Egypt. However, by contrast, WBLP spent comparatively little on research, allocating most of the funds to PZQ distribution. At a total cost of USD153 million over 10 years (1992–2001), the overall prevalence of human *S. japonicum* infection in China was reduced to less than 10% of what it was in the mid-1950s.^19^ This unparalleled progress could not be sustained, however, without eventually reintroducing snail control on a large scale, the original Chinese cornerstone of schistosomiasis control.^20^

The Schistosomiasis Control Initiative (SCI), established in 2002, was based on an initial award of USD34 million from the Bill & Melinda Gates Foundation (BMGF) to assist six African countries, but was later expanded to include several more.^11,28^ Having demonstrated that MDA could be rolled out on a countrywide basis when supported by political commitment and good management, SCI was able not only to enlarge its reach but also to cover other NTDs.^11,29^ The latest important schistosomiasis project before SCORE was the alliance to optimize schistosomiasis control and transmission surveillance in sub-Saharan Africa (CONTRAST), funded for 4 years (2006–2010) by the European Commission at a cost of USD3.5 million, with the mission to optimize schistosomiasis control and transmission surveillance in sub-Saharan Africa. Although relatively small, this multidisciplinary alliance brought together key skills and expertise delivering effective strategies for the long term.^30^

## THE SCORE LEGACY

Responding to perceived gaps in the knowledge base upon which control activities were carried out,^31^ the SCORE program (2008–2019), funded by BMGF at USD23 million, identified key questions, placing them into a common framework geared at producing updated guidelines.^31,32^ Shortcomings with regard to control were thoroughly examined and hard evidence produced for the control and elimination of schistosomiasis. The work was carried out in various epidemiologic settings in eight sub-Saharan countries and represents the largest research program carried out so far.^32,33^ Importantly, the SCORE activities led to durable links between researchers and local control staff, strengthening the likelihood that recommended improvements would be sustained.

Whereas SCI has shown that schistosomiasis morbidity can be kept at bay by well-managed, recurrent retreatment cycles,^11^ SCORE confirmed that MDA alone is not sufficient if the goal is elimination.^32–34^ In fact, the full realization of PZQ’s inability to stem transmission caused (paraphrasing Shakespeare) “the native hue of resolution to change into the pale cast of thought.” Thus, WHO’s 2019 estimate^35^ of “220 million people still lacking treatment,” interpreted as the number of people not yet treated plus those treated but again having become reinfected, tells us that prevalence is far from receding. Even more worrying is that this estimate is based almost entirely on the Kato–Katz stool examination^36^ (for *S. mansoni*) and urine filtration^37^ (for *S. haematobium*), both relatively insensitive assays. These unreliable prevalence data, an unfortunate side-effect of decreased intensity of infection after MDA, can only be remedied by tests of higher sensitivity, for example, polymerase chain reaction,^38^ which, however, requires a laboratory environment. Here, SCORE responded by assessing both the point-of-care schistosome circulating cathodic antigen assay^39,40^ and another assay based on a circulating anodic antigen (CAA).^41,42^ The former is a commercially available, assured POC test, but recommended only for *S. mansoni* infections, whereas the latter recognizes all of the six human schistosome species.^42^ Circulating anodic antigen detection is the most sensitive, assumed to detect single-worm infections, but the test is not yet at the POC level because it includes a sample-preparation step; however, efforts are ongoing to improve its accessibility.^42^ The prospect of detecting several hundred million ultralow intensity infections by large-scale application of high-definition diagnostics promises to be of huge significance, not only because of clinical needs but also because it might paint a revealing picture of ongoing transmission.

The main thrust of SCORE constituted a successful search for situations and practices undermining the efficacy of current MDA strategies to be reflected in improved guidelines. Other research carried out under its aegis initiated and defined new research areas rather than delivering final conclusions, for example, work on persistent hot spots and schistosome population genetics, the former possibly underpinning transmission^43^ and the latter potentially increasing the risk for hybridization and drug resistance.^44^ The abundant SCORE data also enabled the development and testing of predictive models,^45,46^ another potentially fruitful avenue of research. Information as to when and where to apply snail control^47^ constitutes another important area earmarked by SCORE. In this connection, the recent finding that many African snail hosts are nearing their thermo-physiological boundaries^48^ needs urgent investigation, as a major shift in snail geographic ranges could be imminent. Finally, high-definition diagnostics for the detection of infected humans might also be useful for the creation of local transmission indices if combined with reliable snail diagnosis^49,50^ and/or methods for detection of free environmental schistosome DNA.^51^

Some of the goals discussed here are reminiscent of the CONTRAST program,^30^ but what set SCORE apart was its scale and its strategy to coordinate a large number of research teams, converging activities toward common goals, not unlike the more basic work of EMCF 40 years ago.^52^ Naturally, not all important factors could be addressed by SCORE. As outlined in the original proposal,^31^ even some essential subjects, such as female genital schistosomiasis, treatment of children younger than 5 years, mapping of persistent hot spots, and surveillance programs, will have to wait until research has moved forward. However, the wide-ranging data on *S. mansoni* and *S. haematobium* control collected by SCORE are not only representative for the African continent but also of value in consideration of Latin America and the Middle East, and to some extent for *S. japonicum* and *S. mekongi* in the Far East. Notwithstanding that these two species are zoonotic and have amphibious snail hosts, China plans to eliminate schistosomiasis in the near term.^53^ Still, further large-scale studies in the spirit of SCORE would undoubtedly strengthen the worldwide elimination of schistosomiasis, once described as “the most dreadful of the remaining plagues.”^54^
